# A double blind randomized controlled trial comparing primary suture closure with mesh augmented closure to reduce incisional hernia incidence

**DOI:** 10.1186/1471-2482-13-48

**Published:** 2013-10-28

**Authors:** Jeroen Nieuwenhuizen, Hasan H Eker, Lucas Timmermans, Wim CJ Hop, Gert-Jan Kleinrensink, Johannes Jeekel, Johan F Lange

**Affiliations:** 1 Department of Surgery, LUMC, Leiden, The Netherlands; 2 Department of Surgery, Erasmus MC, Rotterdam, The Netherlands; 3 Department of Epidemiology, Erasmus MC, Rotterdam, The Netherlands; 4 Department of Neuroscience, Erasmus MC, Rotterdam, The Netherlands

## Abstract

**Background:**

Incisional hernia is the most frequently seen long term complication after laparotomy causing much morbidity and even mortality. The overall incidence remains 11-20%, despite studies attempting to optimize closing techniques. Two patient groups, patients with abdominal aortic aneurysm and obese patients, have a risk for incisional hernia after laparotomy of more than 30%. These patients might benefit from mesh augmented midline closure as a means to reduce incisional hernia incidence.

**Methods/design:**

The **PRI**mary **M**esh Closure of **A**bdominal Midline Wound (PRIMA) trial is a double-blinded international multicenter randomized controlled trial comparing running slowly absorbable suture closure with the same closure augmented with a sublay or onlay mesh. Primary endpoint will be incisional hernia incidence 2 years postoperatively. Secondary outcomes will be postoperative complications, pain, quality of life and cost effectiveness.

A total of 460 patients will be included in three arms of the study and randomized between running suture closure, onlay mesh closure or sublay mesh closure. Follow-up will be at 1, 3, 12 and 24 months with ultrasound imaging performed at 6 and 24 months to objectify the presence of incisional hernia. Patients, investigators and radiologists will be blinded throughout the whole follow up.

**Disccusion:**

The use of prosthetic mesh has proven effective and safe in incisional hernia surgery however its use in a prophylactic manner has yet to be properly investigated. The PRIMA trial will provide level 1b evidence whether mesh augmented midline abdominal closure reduces incisional hernia incidence in high risk groups.

**Trial registration:**

Clinical trial.gov NCT00761475.

## Background

Incisional hernia (IH) is the most frequently seen long term complication in surgery causing much morbidity and even mortality in patients [1-4]. Despite studies on the optimal closing technique for laparotomies, the risk for IH after midline incision remains about 11-20% [5,6]. In the Netherlands alone about 4000 IH operations are performed each year. Incisional hernia surgery is, in fact, a re-operation to relieve symptoms caused by this common complication and the results of repair are often disappointing [7,8].

Patient-related risk factors for incisional hernia after a laparotomy, like obesity, steroid use, malnutrition, smoking, abdominal aortic aneurysm (AAA), and connective tissue disorders are known [7,9-13]. Despite this knowledge a sufficient method for prevention, has not been developed yet. Most research in the field of incisional hernia surgery has been performed to prevent recurrence after repair. The closure technique of midline incisions has grosso modo remained unchanged since many decades and primarily consists of suturing the linea alba. Interest in prevention of incisional hernias with the aid of synthetic mesh is growing and small, yet promising studies have now been published [14-25].

One specific group of high-risk patients are patients with an AAA. Aortic aneurysm is considered to be related to a type of connective tissue disorder. The connective tissue in these patients is thought to be compromised, playing an important role in the pathogenesis of an aneurysmal distension of the aorta. Healing of the midline fascia after laparotomy may be compromised due to formation of collagen with insufficient strength. Sutures can tear through the fascia and defects can develop in the abdominal wall. The relationship between aortic aneurysm and other abdominal wall hernias, like inguinal hernias, has been reported [26-28]. Retrospective and prospective studies have shown an average risk for incisional hernia after AAA repair of about 30% (Table 1) [9,26,28-34].

**Table 1:** Publications concerning risk for incisional hernia after aortic aneurysm repair with midline incision with a minimum of 2 years follow-up

**Author**	**Year**	**Follow-up**	**Article type**	**# Hernias**	**# AAA**	**%**
Fassiadis et al. [9]	2005	50 months	RCT	20	22	90,9
Rodriguez et al. [29]	2004	36 months	Prospective	14	61	22,9
Liapis et al. [30]	2004	63 months	Prospective	32	197	16,2
Raffetto et al. [28]	2003	33 months	Prospective	50	177	28,2
Augestad et al. [31]	2002	42 months	Case series	49	140	35
Musella et al. [32]	2001	49 months	Prospective	16	51	31,4
Adye and Luna [26]	1998	36 months	Retrospective	18	58	31,0
Holland et al. [33]	1996	24 months	Case series	13	34	38,2
Stevick et al. [34]	1988	38 months	Retrospective	10	27	37,0

Another high risk group is the group of obese patients [5]. Patients with a BMI of 30 or more have a high risk of developing an incisional hernia after midline incision, with an incidence of 22% after 12 months [5,13]. Most recent literature is showing us that even a BMI of more than 27 gives a 20% risk for developing an incisional hernia after midline laparotomy [35]. Considering only 50% of incisional hernia will be clinically evident in the first 12 months, the total incidence is likely to be above 30%. It is known from the study of Burger et al. that an extensive follow up time of up to 10 years is needed to evaluate outcome in hernia surgery [7]. A tailored approach might be necessary, since hernia formation is multifactorial. Thus, the above mentioned high-risk group of patients with obesity and aneurysmal disease can benefit most from prevention.

Some small studies have been performed to evaluate the effect and safety of primary laparotomy wound closure with the aid of prosthetic mesh (Table 2) [14-25]. These studies show a very low risk for incisional hernia and a low infection rate, even when used in contaminated area’s, as seen in colostomy surgery. However, no high quality and adequately powered randomized controlled trial has been performed to evaluate the impact of prophylactic mesh augmentation for prevention of incisional hernia in high risk patients. This is the reason that the **PRI**mary **M**esh Closure of **A**bdominal Midline Wound (PRIMA) trial is being conducted.

**Table 2:** Publications concerning incisional hernia prevention with the aid of prosthetic mesh

**Author**	**Year**	**Type article**	**# Patients**	**Hernia primary**	**Hernia Mesh**	**Follow-up**	**Mesh type**	**Mesh position**
G. Currò et al. [14]	2011	Prospective	95	15/50	2/45	24 months	Polypropylene	Sublay
O. H. Llaguna et al. [15]	2011	Prospective	134	11/62	1/44	17 months	Biological	Intraperitoneal
P. M. Bevis et al. [16]	2010	RCT	85	16/43	5/37	36 months	Polypropylene	Sublay
G. Currò et al.	2010	Prospective	50	8/25	1/25	12 months	Polypropylene	Sublay
M. P. Hidalgo et al.	2010	Cohort	72	-	0/72	46 months	Polypropylene	Onlay
O. H. El-Khadrawy et al. [19]	2009	RCT	40	1/20	3/20	36 months	Polypropylene	Preperitonial
G. Hebert et al.	2009	Cohort	16	-	1/16	6 months	Mix	Sublay
J. Strzelczyk et al. [21]	2006	RCT	74	8/38	0/36	28 months	Polypropylene	Sublay
J.L. O’Hare et al. [22]	2007	Cohort	39	-	1/28	48 months	Polypropylene	Sublay
C. Gutierrez de la Pena et al. [23]	2003	RCT	88	5/44	0/44	36 months	Polypropylene	Onlay
J. Strzelczyk et al. [24]	2002	Prospective	60	9/48	0/12	12 months	Polypropylene	Sublay
A. Pans [25]	1998	RCT	288	41/144	33/144	29 months	Vicryl	Intraperitoneal

### Objective

The objective of this study is to evaluate the effectiveness of incisional hernia prevention in patients after laparotomy for aortic aneurysm and in obese patients with a BMI of more than 27. A double blind randomized controlled trial will compare the commonly used technique of running suture to closure with the aid of a prosthetic mesh.

•The primary outcome measure will be incisional hernia occurrence 2 years postoperatively.

•Secondary outcome measures will cover relevant postoperative complications, post-operative pain and quality of life.

## Methods/design

### Trial design

The trial is a double blinded randomized controlled international multi centre trial comparing traditional closure with running slowly absorbable suture to closure with the aid of prosthetic mesh. A total of 11 centers have agreed to participate in the trial which are located in three different countries (The Netherlands, Germany and Austria). A total number of 460 patients will be included. Patients will be randomized in three groups per-operatively to either receive primary closure, or mesh supported closure either in a sublay or onlay position. Patients will be kept unaware of the procedure until the endpoint of the trial was assessed. Outpatient clinic controls will be done by surgeons or surgical residents blinded for the procedure. Results will be stratificated by center and operation indication.

### Participants

Patients meeting the inclusion criteria scheduled for elective laparotomy will be asked to participate in the study. After ample information has been given, patients will be asked for informed consent.

### Inclusion criteria

•Every elective midline laparotomy for patients with Abdominal Aortic Aneurysm AND/OR patients with a BMI of more than 27^a^.

•Signed informed consent

### Exclusion criteria

•Age < 18 years

•Inclusion in other trials with interference of the primary endpoint

•Life expectancy less than 24 months (as estimated by the treating physician)

•Pregnant women

•Immune suppression therapy within 2 weeks before surgery

•Bovine allergy

### Registration and randomization procedure

Patients who are scheduled for operation and who have given informed consent will be registered by contacting the trial coordinator using the telephone or using the online inclusion randomization system. Included patient are registrated in an online data base (designed and managed by HOVON data center, Rotterdam, the Netherlands) called TOP (Trial Online Process; see http://www.primatrial.nl). The patient name code, date of birth, name of caller, name of responsible physician, sex and eligible criteria will be registered. Every participating institution has its own login code.

Randomisation will take place at the end of the scheduled operation before closing the abdomen in the operating room by contacting the trial coordinator using the telephone or using the online inclusion randomization system. The patient will stay in the randomization group on an intention to treat principle.

### Intervention

Patients will be randomized for three different closing techniques (1A: primary suture closure of the midline fascia, 2B onlay mesh supported closure and 3C sublay mesh supported closure). Both mesh techniques are extensively used in incisional hernia surgery. However, a powered randomized comparison of these two techniques has not been performed. Infection rates in these trials seem low, even in the presence of open bowel [36-41]. Because the study population will not be operated for an incisional hernia, which necessitates extended dissection of the abdominal wall in a previously operated area, infection rates are expected to be lower than the rates mentioned in the literature. Intra-peritoneal placement has not been considered given the high risk for adhesions between viscera and mesh [42].

The mesh will be fixed to the fascia structures with fibrin sealant (Tissucol DUO 500 2,0 ml (Baxter Deutschland GmbH, Unterschließheim, Germany) in order to avoid sutures subcutaneously, to prevent the production of seroma and to simplify the procedure [43]. Nowadays fibrin sealants are occasionally used in inguinal hernia surgery [44-46]. The mesh will be fixed adequately with fibrin sealant to the ventral part of linea alba and posterior rectus sheath. The Optilene Mesh LP, 6 × 35 cm, B. Braun Aesculap AG, Tuttlingen, Germany, will be used as it was shown to have an optimal fixation with fibrin sealant and to provide good tensile strength [47].

Only the first operations of each center will be supervised by one of the PRIMA trial research fellows. If during operation an incisional hernia was discovered the patient was excluded from the trial, as the interest of this study was incisional hernia prevention, not repair. All centers were familiar with the 4:1 suture length to wound length ratio concept although not measured. As the focus of the trial was on the effect of primary mesh augmentation versus common day practice closure, no measurements of the suture closure were done.

#### Group A. Primary closure of the midline

The midline fascia will be closed in all three groups with a running slowly absorbable suture *(MonoPlus, USP 1, Needle HRT48, 150 cm loop, B.Braun Aesculap, Tuttlingen, Germany).* The ratio of suture length to wound length of 4:1 is recommended (but not measured). Subcutaneous tissue and skin are closed in a fashion preferred by the surgeon.

### Mesh supported closure

#### Group B. Onlay mesh supported closure

First, the midline will be closed as indicated in group A.

The Optilene Mesh LP will be positioned on the primary closed midline fascia with an overlap of 3 cm at each side. The mesh will then be fixed with fibrin sealant (5 ml). The fibrin sealent will be applied on the entire surface of the mesh, and in one shot having permanent contact between the mesh and the tip of the joining piece. Immediately after application of the fibrin sealant, the mesh will be smoothed with the back of a forceps to get a good fixation of the mesh on the entire surface and especially on the suture line. If present, it is also possible to use spray fixation using the EASYSPRAY system, *Deutschland GmbH, Unterschließheim, Germany.* When laparotomy is larger then 25 cm use 2 applicators of Tissucol (10 ml). Subcutaneous tissue and skin are closed in a fashion preferred by the surgeon.

#### Group C. Sublay mesh supported closure

A space will be created between both posterior rectus sheaths and the rectus muscle. Both posterior rectus sheath edges are sutured using a running slowly absorbable suture,*(Monoplus, USP1, Needle HRT48, 150 cm, B. Braun Aesculap AG, Tuttlingen, Germany)*. A suture length to wound length ratio of 4:1 was recommended (not measured). The Optilene Mesh LP will then be placed between the posterior rectus sheath and the rectus muscle with an overlap of 3 cm at each side and fixed with fibrin sealant (5 ml). The fibrin sealent will be applied on the entire surface of the mesh, in one shot having permanent contact between the mesh and the tip of the joining piece. Immediately after application of the fibrin sealant, the mesh will be smoothed with the back of a forceps to get a good fixation of the mesh on the entire surface and especially on the suture line. If present, it is also possible to use spray fixation using the EASYSPRAY system, *Deutschland GmbH, Unterschließheim, Germany.* When laparotomy is >25 cm use 2 applicators of Tissucol (10 ml). The midline anterior rectus sheath will be closed using a running slowly absorbable suture *(Monoplus, USP1, Needle HRT48, 150 cm, B. Braun Aesculap AG, Tuttlingen, Germany)*, covering the mesh. A suture length to wound length ratio of 4:1 was recommended (not measured). Subcutaneous tissue and skin will be closed in a fashion preferred by the surgeon.

#### Postoperative treatment

Wound drainage will not be routinely applied. Seromas do not have to be punctured or drained, but can be left untreated to resolve spontaneously.

### Implementation

#### Pre-operative data

•Date of birth

•Length and weight

•Smoking history (current smoker ( Y or N )

•Medical history (COPD, diabetes, cardiac disease)

•Preoperative Radiotherapy or chemotherapy

•Preoperative corticosteroids

•Postoperative corticosteroids

•Previous abdominal operations

•Other abdominal hernias (inguinal, umbilical, epigastric hernias)

•ASA class

•Width of linea alba (when pre-operative C.T. imaging is available)

•Size of aneurysm and location

•Epidural catheter

#### Operation data

•Type of operation

•Type and length of prosthesis

•Volume of fibrin sealant applied

•Length of incision

•Blood loss

•Operation time

•Antibiotic prophylaxis

•Suture material

•Drains and location

•Thrombosis prophylaxis

•Pain medication

•Complications (intestinal lesions, bleeding, other)

#### Post-operative data

•Blood transfusion

•Postoperative ventilation and duration

•Postoperative ileus and duration

•Postoperative complications:

○ Surgical Site Infection, according to the guidelines proposed by Mangram in 1999 [48]. (Appendix)

○ Wound hematoma: accumulation of blood in the wound area, which warrants surgical exploration and intervention.

○ Seroma subcutaneously

○ Pulmonary infections

○ Ventilation problems

○ Re-intervention and difficulties caused by the mesh at re-entry

○ Re-admission and indication

#### Ultrasound examination

At 6 and 24 months ultrasound imaging will be performed to examine the midline for any asymptomatic clinically not detectable incisional hernias. This will provide valuable information about the onset of an incisional hernia. Size and location of all incisional hernias noted radiographically will be registered, as well as complaints presented by the patients. Endpoint of this study will be at 2 years follow up. At this follow-up the presence of a hernia will be investigated by physical examination and ultrasound imaging.

#### Outpatient follow-up

The follow-up schedule is displayed in Table 3. During visits the following information will be gathered.

•Outpatient clinic visit at 1, 3, 12 and 24 months

○ Incisional hernia

○ Wound infection

○ Seroma formation

○ Other wound problems

○ Inguinal hernia

•Ultrasound at 6 and 24 months

•VAS score at 1 month

•VAS scores and Quality of Life forms preoperatively ( day of operation or the day before) and at 3, 12 and 24 months

**Table 3:** Follow up schedule

**Evaluation moments**	**MOS SF-36 (1)**	**EQ-5D (2)**	**VAS score (3)**	**Outpatient clinic**	**Ultrasound**
Pre-operative	X	X		X	
1 month			X	X	
3 months	X	X	X	X	X at 6 months
12 months	X	X	X	X	
24 months	X	X	X	X	X

(1) MOS SF-36: Questionnaire concerning quality of life (SF-36 ™ Health Survey, Medical outcomes Trust, Boston, Massachusetts 02116, USA).

(2) EQ-5D: Euro Qol Group quality of life questionnaire.

(3) VAS score: Pain measurement tool on which patients can define their pain on a sliding scale.

### Economical evaluation

Cost effectiveness will be calculated after 2 years. The direct costs, admissions, operation costs, costs of materials and treatment of complications and incisional hernias, will be calculated. Quality Adjusted Life Years will be calculated.

An incisional hernia correction costs Є3777,-. When 100 patients are operated with the aid of a mesh insertion we estimate to prevent 15 incisional hernias (=Є56.655,-). One hundred meshes cost approximately Є30.000,-. We would save Є26.655,- if all incisional hernias are repaired. We did not include all extra costs as for example visits to the general practitioner, but these will be included in our final analysis.

### Statistical analysis

Three comparisons will be made leading to pair-wise comparison at alpha = 0.017 (=0.05/3) according to Bonferroni’s correction for multiple testing. Assuming a 30% rate of incisional hernia in group A, and about 10% in both groups B and C, for a power of 90% comparing group A versus group B and C, 92 patients are required in group A and 164 in groups B and C. Allowing for some dropouts, 100 will be included in the control group and 180 in each experimental group.

It is expected that differences between groups B and C can only be demonstrated with a very large number. Therefore it was decided to set the objective to showing “non inferiority” for onlay (group C) versus sublay (group B). Setting the non-inferiority margin at 10%, the power to show non-inferiority regarding the incidence rate of incisional hernia will be greater than 80%.

For the comparison of both experimental groups with the control group, Kaplan-Meier curves will be constructed and the log-rank tests will be performed. These logrank tests will be done with stratification by center and operation indication.

For the comparison of both experimental groups B and C, the cumulative 2-years probability will be calculated with the one-sided 98.3% confidence interval for the difference. Analysis will be done according to the intention-to-treat principle in comparing group A with groups B and C. For the comparison of groups B and C a per-protocol analysis will be the primary analysis.

Comparison of VAS and QOL scales between groups will be done using Repeated Measures Anova (SAS PROC MIXED) with baseline value, age, gender, operation indication and center as covariates.

The following putative risk factors regarding incisional hernia (smoking, infection, diabetes, corticosteroids) will be evaluated using Cox-regression.

#### Serious Adverse Event (SAE) reporting & Monitoring

A SAE will be reported to the Dutch Department for Human Research (Centrale Commissie Mensgebonden Onderzoek), Baxter and Braun within 24 hours.

Requirements for SAE reporting will be:

1. (Prolonged) Hospitalisation (difined as a longer stay in the hospital than normally expected caused by a postoperative complication)

2. (Re-)operation

3. Death

Once a year, data from each center will be monitored. In compliance with GCP guidelines, monitors will verify data collected on data collection forms against source documents. Source documents are defined as any original records or data related to the trial or to subject treatment or medical history. Source documents include: original hospital, clinical, and office charts, laboratory notes, subject diaries or evaluation checklists, pharmacy records, recorded data from automated instruments, transcriptions (certified to be accurate after verification), magnetic media, or x-rays.

### Ethics

Before centers could participate in this trial, approval was obtained from the local medical ethics committee (Medische Ethische Toetsings Commissie, Erasmus MC). Patients will be extensively informed about the research project and can only participate after giving informed consent. Patients will always be permitted to withdraw from the study without providing further reasons. This will have no consequences for further treatment. Data of these patients will be evaluated in the final analysis. This trial was registered at Clinical trial.gov under NCT00761475.

### History and current status

After Ethical approval was obtained the trial started including patients in the middle of 2009. Initially the intake of patients was rather slow. This was attributed to the the low number of participating hospitals, the continued increase of laparoscopy and endovascular treatment, and the inclusion criteria of BMI >30. After the publication of Seiler et al. the BMI inclusion criteria were lowered from 30 to 27 [35]. The BMI amendment and the inclusion of additional participating hospitals made it possible to include more patients per month. Currently the trial is in the final stage of the inclusion of patients. It is estimated that the last patients will be seen in the outpatient clinic in the beginning of 2015. Around this time the final results will be subjected to peer-review for publication.

## Discussion

Incisional hernia continues to be one of the most frequent complications after laparotomy. Up to this date no intervention strategy has led to a resolution to this problem. In high risk patients, with a risk for incisional hernia more than 30%, an alternative technique with lower incisional hernia incidence would be highly desirable.

In daily practice almost all midline laparotomies are closed with slowly absorbable running sutures. This technique seems ample for low risk patients. Despite the high incidence of incisional hernia, this technique is still used in high risk patients. These patients are known to have altered collagen synthesis in wound repair or increased abdominal wall stress, leading to insufficient repair of the midline after operation.

In incisional hernia surgery the use of prosthetic mesh has proven its effectiveness and safety. For this reason a RCT investigating the effectiveness and safety of augmenting the closure of the midline with prosthetic mesh in high risk patients is being conducted. A high level of evidence will be obtained due to the design of the study, as it was a randomized, double blind, powered, multicenter study.

## Conclusion

The PRIMA trial is a prospective international multicenter double blind randomized trial comparing primary suture closure of midline laparotomy to closure aided with a prosthetic mesh. This trial might provide the surgical society a technique to prevent incisional hernia in high risk patients.

## Endnote

^a^The initial inclusion criteria featured patients with a BMI of 30 or higher. However as stated before, a study was published during the enrolment of this trial demonstrating that patients with a BMI 27 or more could also be included [35]. We amended our protocol to lower our inclusions criteria for BMI, from 30 to 27.

## Appendix

**Criteria for defining a Surgical Site Infection (SSI)**[49]

*Superficial Incisional SSI*

Infection occurs within 30 days after the operation *and* infection involves only skin or subcutaneous tissue of the incision *and* at least *one* of the following:

1. Purulent drainage, with or without laboratory confirmation, from the superficial incision.

2. Organisms isolated from an aseptically obtained culture of fluid or tissue from the superficial incision.

3. At least one of the following signs or symptoms of infection: pain or tenderness, localized swelling, redness or heat *and* superficial incision is deliberately opened by surgeon, *unless* incision is culture-negative.

4. Diagnosis of superficial incisional SSI by the surgeon or attending physician.

5. Do not report the following conditions as SSI:

6. Stitch abscess (minimal inflammation and discharge confined to the points of suture penetration).

7. Incisional SSI that extends into the fascial and muscle layers (see deep incisional SSI).

## *Deep incisional SSI*

Infection occurs within 30 days after the operation if no implant is left in place or within 1 year if implant is in place and the infection appears to be related to the operation *and* infection involves deep soft tissue (e.g., fascial and muscle tissue) of the incision *and* at least *one* of the following:

1. Purulent drainage from the deep incision but not from the organ/space component of the surgical site.

2. A deep incision spontaneously dehisces or is deliberately opened by a surgeon when the patient has at least one of the following signs or symptoms: fever (>38°C), localized pain, or tenderness, unless site is culture negative.

3. An abscess or other evidence of infection involving the deep incision is found on direct examination, during re-operation, or by histopathologic or radiological examination.

4. Diagnosis of a deep incisional SSI by a surgeon or attending physician.

Notes:

1. Report infection that involves both superficial and deep incision sites as deep incisional SSI.

2. Report an organ/space SSI that drains through the incision as a deep incisional SSI.

## *Organ/Space SSI*

Infection occurs within 30 days after the operation if no implant is left in place or within 1 year if implant is in place and the infection appears to be related to the operation *and* infection involves any part of the anatomy (e.g., organs or spaces), other than the incision, which was opened or manipulated during an operation *and* at least *one* of the following:

1. Purulent drainage from drain that is placed through a stab wound into the organ/space.

2. Organisms isolated from an aseptically obtained culture of fluid or tissue in the organ space.

3. An abscess or other evidence of infection involving the organ/space that is found on direct examination, during reoperation, or by histopathologic or radiologic examination.

4. Diagnosis of a deep organ/space SSI by a surgeon or attending physician.

## Competing interests

This study was supported by B. Braun Aesculap GmbH, Tuttlingen, Germany and Baxter Deutschland GmbH, Unterschließheim, Germany. None of these sponsors were involved in the design, conduct or analysis of the study. Disclosure: The authors declare no other conflict of interest.

## Authors’ contributions

JN drafted the original protocol and wrote the protocol manuscript, HHE drafted following procotol amendments and was involged in patient inclusion and data gathering, LT drafted following procotol amendments and was involved in patient inclusion and data gathering, WCJH is the trial statistician and performed the power analysis and was responsible for the trial methodology, GJK was involved in writing of the first manuscript and all following amendments, JJis initiator and creator of the PRIMA trial and was involved in writing of the first manuscript and all following amendments, J. F. Lange is initiator and creator of the PRIMA trial and was involved in writing of the first manuscript and all following amendments, PRIMA Trialist group were all responsible for including patients, organizing and conducting follow-up. All authors read and approved the final manuscript.

## Pre-publication history

The pre-publication history for this paper can be accessed here:

http://www.biomedcentral.com/1471-2482/13/48/prepub
